# The Danger of Salvarsan

**Published:** 1911-03

**Authors:** 


### The Danger of Salvarsan

SINCE the announcement that Erlich had discovered in his 606th combination a definite cure of syphilis and additional information was given to the public that one injection was sufficient to eradicate the disease, the newspapers and magazines have devoted more than the usual amount of space given to scientific matters and have builded up a pyramid of hope for those afflicted with this most ancient of diseases. There followed the period of waiting while investigators experimented with the preparation and the eager watchfulness for reports from the German clinics where much of the preliminary work was done. In the usual course of events, and on schedule time, the preparation was placed on sale in the United States shortly after January 1, 1911. Since then it has been used with lavish hand in all sorts of conditions and in almost every phase of the disease which has been discovered or imagined since the days of those early Chinese observers who 5,000 years before Christ penned graphic, if crude, descriptions of the various lesions and outlined the treatment by mercurial inunction.

That there are physical dangers in the indiscriminate use of salvarsan is well known; there has been death following its administration and serious eye disturbances; yet the mere physical disabilities and even the danger of death in isolated cases is not the real danger which lies at the door of the drug. Its unwarranted acceptance as an absolute specific has been drilled into the lay mind through newspaper channels—and at the hands of those of the profession who seek the ready dollar at the expense of professional self respect. It has been accepted by the public as the one, the only avenue of escape from those terrors of late lesions which have been the haunting nightmare of the luetic and there is cause for sincere regret that the medical profession has placed itself in a position of blindly recommending the use of the preparation without sufficient proof that it was all that was claimed for it. There are coming into print through the medical journals in all parts of the world, most notably in Germany and Russia, reports which indicate that because of the large number of relapses of the disease salvarsan is not an absolute specific and that one or even repeated injections do not cure syphilis. There is little doubt that its action is beneficial; that it does clear up the visible manifestations of the disease in relatively short time in some cases and that the Wasserman reaction is negative in relatively a few hours after its administration. The same is true of mercury.

The menace of salvarsan lies not in the damage which it may do when improperly administered to the individual who receives it. Its danger lies in the false sense of security which its ready acceptance has bred in the lay mind and it requires little more than ordinary human intelligence to foresee the evils which must necessarily follow in its wake. One injection retards the disease and the simple, misguided, public little given to intimate reasoning along any line, much less in matters of scientific trend, believes itself wholly cured. There is removed from the public mind the necessity for care in sexual relations or delay in assuming marital responsibilities. The inevitable result, unless there be a more serious consideration of the uses and limitations of the preparation, will be the birth within the next five or ten years of children who will bear the indelible mark of inherited syphilis and there shall be thrust upon us and our responsibilities—through our own misguided enthusiasm—a brood of immaturities with the mental or physical defects which are the handmaidens of syphilis.

The fact that salvarsan is an arsenic preparation of high activity is apparently being ignored by many of those who are using it as a routine treatment for syphilitic infection. Then, too, there appears to be a woeful lack of knowledge concerning the results of administering less than a maximum dose in any given case and on this is builded the vast structure of misplaced confidence which is being reared day by day and giving open door to the chief danger of misuse of the drug. It is a well established fact that the fundamental principle upon which rests the whole value of the salvarsan is the administration of a large amount of arsenic—a maximum dose—which is supposed to destroy the organisms. Less than the maximum dose destroys some; those remaining become what is known as "arsenic fast" and are not affected even by repeated administrations of larger amounts. It is here that the initial danger lies for these arsenic fast organisms have power to and do bring about the relapses which are being reported.

This is in no sense a condemnation of salvarsan. It is simply a serious and calculated note of warning, a plea for less enthusiastic acceptance for freehanded use of a drug which has distinct merit in selected cases but whose general use has limitations which are well defined. We have not yet reached the time when syphilis may be wiped away with a magic sweep. Salvarsan is useful—but it is not without danger and the danger lies not with the ones of today nor of those of tomorrow, but with those who are to come after; the innocent, helpless victims of loose-footed affection and the careless administration of a misused drug.

Salvarsan is useful in the treatment of syphilis; and its greatest usefulness is most apparent when it is followed by a thorough course of mercury.

